# Information interaction and social support: exploring help-seeking in online communities during public health emergencies

**DOI:** 10.1186/s12889-023-16151-3

**Published:** 2023-06-27

**Authors:** Yanni Yang, Yue Zhang, Anling Xiang

**Affiliations:** 1 School of Literature and Media, China Three Gorges University, Yichang, 443000 China; 2 David Nazarian College of Business and Economics, California State University Northridge, Los Angeles, 91330-8378 USA; 3 School of Journalism and Communication, Minzu University of China, Beijing, 100081 China

**Keywords:** Public health emergency, COVID-19 pandemic, Online community, Online help-seeking, Information interaction, Online social support

## Abstract

**Background:**

During public health emergencies, online community users can obtain social support and assistance through information interaction in the online community. This study takes the COVID-19 pandemic as the context and aims to analyze the influence of user information interaction in online communities on the acquisition of social support during this public health emergency.

**Methods:**

Data collected from help-seeking posts in the “COVID-19 Patients Help-Seeking Dialog” subforum on China’s Sina Weibo were used as the research sample. The influence of the frequency of interaction and responsiveness on help seekers’ receipt of online social support was analyzed, and the moderating effect of help seekers’ identity type and intensity of online community use was explored.

**Results:**

The results reveal that the frequency of interaction positively impacts informational support (β = 0.367, *p* < 0.001) and negatively impacts emotional support (β=-0.240, *p* < 0.001), and the responsiveness of other users toward help-seeking posts positively impacts emotional support (β = 0.145, *p* < 0.01). Moreover, help seeker’s identity type and intensity of online community use significantly moderate the relationship between the frequency of interaction and the emotional support obtained by the help seeker.

**Conclusions:**

The study highlights the impact of user information interaction on obtaining help-seeking information from online communities for social support. The initiative would facilitate the resolution of issues related to users’ information help-seeking during public health emergencies.

## Background

Major public health emergencies have significant impacts on a country’s economy and society, which often lead to dramatic changes including, in the digitally connected world today, forcing people to work and conduct life activities through remote modalities, facilitated by online communities. As important venues for online information interaction, online communities not only provide platforms for users to receive current information (such as news) and to express opinions but also serve as important channels to disseminate help-seeking information [1]. Online communities provide various types of information channels for help seekers as a supplement to official authority. During public health emergencies, various online communities, to increase information dissemination, have taken various measures to organize relevant topics and enhance information service to community users; these measures include labeling certain hot topics, recommending popular posts, and organizing discussion sections. In China, given the influence of the COVID-19 pandemic since the spring of 2020, the “Coronavirus Disease 2019 (COVID-19) Patients Help-Seeking Dialog” subforum on Sina Weibo is an example of these efforts. Sina Weibo is a representative online community platform in China that is somewhat similar to Twitter. As the largest online community platform in China, Sina Weibo has 586 million monthly active users and 252 million daily active users by the end of 2022 [2]. As of May 2023, the subforum’s posts have been viewed 5 billion times and have 504,000 followers [3].

Throughout the COVID-19 pandemic, online community users have posted help-seeking messages, and other users have reposted and commented on those help-seeking messages, constituting information interaction among users. During the information interaction process, help seekers obtain valuable information (e.g., other users in the online community provide plasma supply information or information about hospitals where COVID-19 patients could be treated); help seekers can also express their mood, share emotions, and arouse public sympathy in the online community [4]. Help seekers intend to have their help-seeking information disseminated and hope to receive material assistance or emotional support [5]. That is, help seekers in online communities obtain online social support through information interaction. Social support is a broad concept that comprises various types of support, such as informational support, emotional support, instrumental support, and social member support [6]. As informational and emotional support are also the most focused dimensions in previous studies [7, 8], this study mainly examines help seekers’ receipt of online social support from the perspectives of informational and emotional support. Informational support includes providing facts, guidance, or advice, while emotional support involves expressions of empathy, concern, caring, love, and trust [9].

Concerning help-seeking in public health emergencies, Asril et al. [10] explored the predictors of help-seeking behavior among Indonesian adults, Zhou et al. [4] discussed the relationships between the features of help-seeking posts and assistance and Liu et al. [11] analyzed the influence of individual support-seeking strategies on the social support provided. As discussed above, although previous studies focus on users’ receipt of social support in public health emergencies from the perspective of help-seeking content posted on social media, there has been insufficient attention on users’ information interaction regarding help-seeking during public health emergencies. Less is known about how information interaction in online communities influences help seekers’ receipt of social support during public health emergencies such as COVID-19.

In recent years, the impact of user information interaction on social support has attracted much attention. High information interaction experiences are typically determined by high reciprocity rates [12]. During information interaction in an online community, users obtain benefits from the interaction process. Previous studies indicate that information interaction among users in an online community is closely related to social support [13]. When there are limited in-person communication opportunities, online interactions help people seek social support [14].

Therefore, this study takes the COVID-19 pandemic as the context and analyzes the influence of users’ help-seeking information interactions in online communities on the acquisition of social support, which provides a new perspective regarding help-seeking during public health emergencies, so as to fill the gap and further expand the relevant research. We believe that the study’s findings would have significant practical implications for explaining the emotional and informational support from online communities when facing a public health emergency, particularly its impact on alleviating the isolation people experienced due to quarantine, and shedding light on public health policy improvement.

## Hypothesis development

The frequency of interaction, which refers to the number of times the help seeker interacts with other users, is an important dimension for measuring user interactions [15, 16]. During the COVID-19 pandemic, help seekers post messages in online communities for help; if they frequently interact with others, other users will obtain more information about the nature of help requested, such as the help seeker’s extent of difficulty, extent of urgency, or degree of danger faced. As a result, the willingness of other users to provide social support will be stronger; those other users will then be more willing to repost (retweet) or comment on the help-seeking message, or offer consolation/encouragement or other emotional support in the comments section under the original post. During real-time online communication, frequent information interaction in online communities helps to build strong connections among users in a weak network relationship [17]. Information interaction among online community users provides direct or indirect help to the help seeker; on the other hand, such interaction provides emotional comfort to the help seeker through comments on the original post, which increases the help seeker’s chances of receiving both informational and emotional support. Based on the above discussions, this study proposes the following hypotheses:

H1: The frequency of interaction between the help seeker and other users positively impacts the help seeker’s receipt of social support;

H1a: The frequency of interaction between the help seeker and other users positively impacts the help seeker’s receipt of informational support;

H1b: The frequency of interaction between the help seeker and other users positively impacts the help seeker’s receipt of emotional support.

Responsiveness, which refers to the degree to which users in the online community timely respond (such as forwards, comments, etc.) to help-seeker’s posts, is another important dimension for measuring user interactions in online communities [18]. Responsiveness is critical to help seekers who attempt to obtain timely information in online communities [4]. People learned about event-related trends through online communities during the COVID-19 pandemic. When users find help-seeking information while browsing, they often respond in a timely manner; responses include reposting (retweeting) the help-seeking messages, providing the sought-after information, or offering emotional support regarding the original messages. Upon the help seekers perceiving other users’ prompt attention and active responses, his/her stress, anxiety, neediness, and other troubling feelings can be alleviated. Studies on the interactive behavior of online community users indicate that users’ perceived responses from other users affects his/her attitudes toward the use of online communities and their relationships with other users [19]. Previous studies have also shown that the prompt responses of users’ friends on Facebook play an important role in their social support acquisition [20]. Therefore, we argue that when a member of an online community posts a message seeking help, the responsiveness of other users has an impact on the help seeker’s receipt of social support. This study proposes the following hypotheses:

H2: The responsiveness of other users toward the help-seeking posts positively impacts the help-seeker’s receipt of social support;

H2a: The responsiveness of other users toward the help-seeking posts positively impacts the help-seeker’s receipt of informational support;

H2b: The responsiveness of other users toward the help-seeking posts positively impacts the help-seeker’s receipt of emotional support.

Additionally, we focus on the impact of celebrity identity in the online community on the celebrities themselves’ online social support acquisition. Ledbetter and Redd suggest that fans’ perceptions of interactions with celebrities reduces uncertainties in communication and reinforces fans’ trust and loyalty, which enhances celebrities’ receipt of social support [21]. Online opinion leaders are usually at the center of information dissemination in online communities, and they frequently interact with other users in communities. Online opinion leaders not only act as a bridge for information dissemination but also obtain more social support, whether informational support or emotional support [9]. During public health emergencies, opinion leaders or general users in the online community can post information for help and obtain social support from other users. This study explores and compares the impact of help seeker’s identity types (ordinary users and opinion leaders) on online social support acquisition. That is, the positive moderating effect of help seeker’s identity types on the influence of information interaction on help seeker’s receipt of social support is investigated. Based on the above analysis, this study proposes the following hypotheses:

H3: The differences in the identity type of the help seeker in the online community positively moderate the influence of the information interaction between the help seeker and others on the help seeker’s receipt of social support;

H3a: The differences in the identity type of the help seeker positively moderate the influence of the frequency of interaction between the seeker and others on the help seeker’s receipt of informational support;

H3b: The differences in the identity type of the help seeker positively moderate the influence of the frequency of interaction between the seeker and others on the help seeker’s receipt of emotional support;

H3c: The differences in the identity type of the help seeker positively moderate the influence of the responsiveness of other users toward the help-seeking post on the help seeker’s receipt of informational support;

H3d: The differences in the identity type of the help seeker positively moderate the influence of the responsiveness of other users toward the help-seeking post on the help seeker’s receipt of emotional support.

In addition to identity type, help seekers’ receipt of social support in the online community is also significantly impacted by the help seeker’s intensity of online community use. Smailhodzic et al. [22] found that the most common type of social media use by patients for health-related reasons is social support. Lee and Cho [23] examined the relationships among online community use, social support, and depression among people with movement or mobility disabilities in Korea; they confirmed that the intensity of online community use significantly predicted informational support. Frison and Eggermont [24] found that girls who actively use Facebook perceive online social support and benefit from actively using this social media. Regardless of whether the help seeker has engaged in an online community in the past, during public health emergencies, help seekers post in online communities to obtain social support. To explore the intensity of online community use of help seekers, its influence on social support, and the positive moderating effect of use intensity on the association between information interaction and social support, this study proposes the following hypotheses:

H4: The differences in intensity of online community use positively moderate the influence of the information interaction between the help seeker and others on the help seeker’s receipt of social support;

H4a: The differences in intensity of online community use positively moderate the influence of the frequency of interaction between the seeker and others on the help seeker’s receipt of informational support;

H4b: The differences in intensity of online community use positively moderate the influence of the frequency of interaction between the seeker and others on the help seeker’s receipt of emotional support;

H4c: The differences in intensity of online community use positively moderate the influence of the responsiveness of other users toward the help-seeking post on the help seeker’s receipt of informational support;

H4d: The differences in intensity of online community use positively moderate the influence of the responsiveness of other users toward the help-seeking post on the help seeker’s receipt of emotional support.

**Fig. 1:**
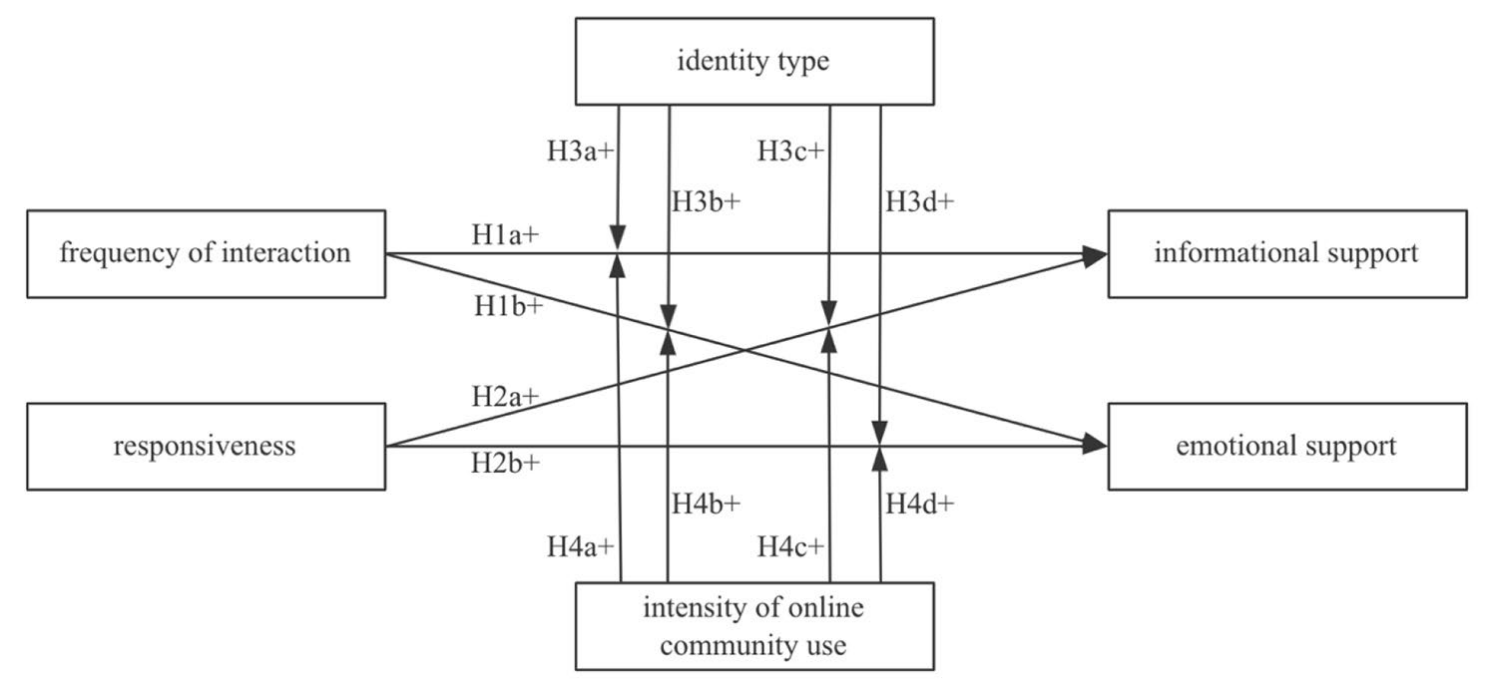
Research model

Based on the above analysis, this study focuses on the dynamics of the interaction between help seekers and other users and the help seekers’ receipt of social support; and draws conclusions to construct a model, as shown in Fig. 1.

## Materials and methods

### Data collection

In the challenging time of the COVID-19 pandemic in China, online platforms organized venues to facilitate help-seeking. On January 29, 2020, the leading Chinese online community, Sina Weibo, opened the “COVID-19 Patients Help-Seeking Dialog” subforum. Then, after the initial full-scale lockdown, the Chinese government partially reopened some critical services; as of March 24, 2020, some hospitals in Hubei Province resumed normal outpatient clinics, and patients could make direct appointments for doctor’s office visits and treatment visits. During the time period between the opening of the COVID help subforum and the resumption of outpatient clinics, tens of thousands of help-seeking messages were posted on the subforum. After verification by Sina Weibo, more than 3,000 messages were forwarded to government authorities; furthermore, those help-seeking messages on this subforum were read more than five billion times. The data generated via user information interactions on this subforum were crawled and analyzed to explore online community users’ help-seeking behaviors and help-seekers’ receipt of social support.

This study collected 807 help-seeking posts from the “COVID-19 Patients Help-Seeking Dialog” subforum on Sina Weibo (hereafter referred to as the “Weibo COVID subforum” or “Weibo subforum”) that were posted between January 29, 2020 and March 24, 2020, as well as the data from 59,244 comments and 85,670 reposts related to the 807 original posts. The data included the help seekers’ account information and the content of the help-seeking posts, the time and content of each comment to or repost of each original help-seeking post, and the help seeker’s responses to comments and reposts.

Data were mainly collected utilizing the Qingbo Analysis System (QAS) (http://www.gsdata.cn), which is an analytics platform that utilizes algorithms with natural language processing and is deemed one of the top social media analysis systems in China [25]. In this study, we used the Qingbo Analysis System to collect and analyze the information on Sina Weibo and employed a statistical analysis function for data analysis. The website of the data to be crawled was put into QAS and the raw dataset was obtained after the crawling was completed. The data are freely available in the public domain on request and do not involve privacy and sensitive personal information. To eliminate invalid, redundant, and erroneous data from the raw data, upon collection of the help-seeking posts and their comments/reposts/responses, we cleaned and processed the data. Afterward, we reorganized the dataset and converted it into SPSS identifiable data.

### Variable measurement

To measure and analyze the online interaction data collected above, we took references from the following studies: Wang [26] used the duration and frequency of online interactions to measure online interactions, and Li et al. [27] measured the frequency of interaction between online users based on the number of interactions. Based on previous studies, in the analysis of online interactions between help seekers and other users, this study mainly uses the number of times a help seeker interacted with other commenters or reposters to measure the frequency of interaction between the help seeker and other users; to measure other users’ responsiveness toward a help seeker, we mainly use the time lag between the original post and the comments to or reposts of that original post.

Each response from the help seeker to other users’ reposts of and comments on the original help-seeking post is counted as one interaction; the total number of responses by the help seeker to a repost or comment on the original help-seeking post is denoted by N; the number of reposts and comments under the original help-seeking post is denoted by M; then, the frequency of interactions between the help seeker and commenters or reposters is denoted by F.

1 $$F=\sum _{i=1}^{M}{N}_{i}$$

For each original help-seeking post, the time lag between each repost or comment (i) and the original help-seeking post is calculated and denoted by T. The average time lag between the original help-seeking post and reposts and comments is represented by ET.

2 $$ET={\sum }_{i=1}^{M}{T}_{i}/M$$

Then, for each original help-seeking post, the responsiveness of other users (R) is calculated by subtracting the help-seeking post’s average time lag from the maximum average time lag.

3 $$R = maxET - ET$$

As the focus of this paper is to examine help seekers’ receiving online social support from the perspective of information interaction among online community users, social support is measured according to the categories of informational support and emotional support. Chen et al. [28] measured the receipt of social support in online health communities in terms of informational support and emotional support and used the number of responses that offer informational support and emotional support, respectively, as the indicators for the two types of support. Hayes et al. [29] proposed that when users post messages on social media for help and receive more “likes,” “upvotes,” or “+1s,” the help seekers perceived more social support. In user information interaction, the social support that help seekers receive from the online community is mainly reflected through more users reposting and commenting on their help-seeking posts [4]. Based on the above analysis, this study uses the number of reposts of and comments on the original help-seeking post in the online community as the indicator of the informational support received by a help seeker. Moreover, the emotional value of the interaction content among the help seeker and other users indicates the degree of emotional support received by the help seeker. Thus, this study uses the emotional value of the interactive content associated with the help seeker’s post as the indicator of emotional support.

The number of reposts of and comments on the original help-seeking post is designated as the value of informational support (S); the number of reposts of the original help-seeking post is denoted by R; and the number of comments on the original help-seeking post is denoted by C. Then,

4 $$S = R + C$$

Emotional support is calculated from the emotional value of the text interactions between the help seeker and other users associated with the help seeker’s original post. The emotional value of a text content *j* through which the help seeker interacts with others is denoted as *e*, and the total number of texts that the helper interacts with others is *W*. Then, the value of emotional support *E*_*W*_ is expressed in Eq. (5).

5 $${E}_{W}=\frac{{\sum }_{j=1}^{W}{e}_{j}}{W}$$

Furthermore, this study considers the moderating effect of help seekers’ characteristics on the relationship between their interactive behavior and receipt of online social support. The first characteristic is the help seeker’s identity type, which is classified by the help seeker’s influence on Weibo. The other characteristic is the help seeker’s online community use intensity, which is measured by the help seeker’s intensity of Weibo use [30].

The emotional value of text content, the help seeker’s influence on Weibo, and the help seeker’s intensity of Weibo use are calculated using the Qingbo Analysis System. In this system, the calculation method of the help seeker’s influence on Weibo and the help seeker’s intensity of Weibo use were proposed by relevant studies [31]. The help seeker’s identity type is categorized into those with low influence and those with high influence. The help seeker’s intensity of online community use is categorized into those with low intensity of online community use and those with high intensity of online community use.

### Data analysis

To verify the hypothesis regarding the influence of information interactions between users on a help seeker’s receipt of online social support, based on descriptive statistical analysis, statistical regression analyses between variables are carried out. SPSS (version 21.0, IBM Corp) was employed to conduct descriptive statistical analysis and regression analysis. To examine the impact of information interactions between help seekers and others in online communities on the receipt of social support and to examine the moderating effect of help seekers’ identity type and their intensity of online community use, this study applied the method proposed by Baron and Kenny [32] while testing the moderating effects. Centralized transformation and standardization were carried out for the independent, dependent, and moderating variables. All statistical analyses were carried out with p < 0.05 as the threshold for statistical significance.

## Results

### Descriptive statistics analysis

A descriptive statistics analysis was performed for the main variables before regression analysis (Table 1). During the COVID-19 pandemic, help seekers who posted information for help in the Weibo COVID subforum engaged in a large number of information interaction activities with others. Help seekers interacted with other users 3.03 times on average, and the mean value of responsiveness was 22.41. The mean value of the informational support obtained by each help-seeking post, that is, the mean value of the number of reposts and comments on the help-seeking post, was 501.35. The mean value of emotional support, that is, the mean emotional value of the content that the help seeker interacted with others, was − 9.98. The results indicate that the frequency of interaction has a statistically significant positive correlation with informational support (r = 0.368, p < 0.01) and a significant negative correlation with emotional support (r=-0.282, p < 0.01) and that responsiveness has a statistically significant positive correlation with emotional support (r = 0.144, p < 0.01) but a nonsignificant correlation with informational support.

**Table 1:** Descriptive statistics and correlation coefficients of the main variables

	M	SD	1	2	3	4
1. Frequency of interaction	3.03	3.00	1			
2. Responsiveness	22.41	2.04	-0.020	1		
3. Informational support	501.35	764.92	0.368**	-0.040	1	
4. Emotional support	-9.98	17.81	-0.282**	0.144**	-0.088	1

Notes: ** p < 0.01

### Regression analysis

**Fig. 2:**
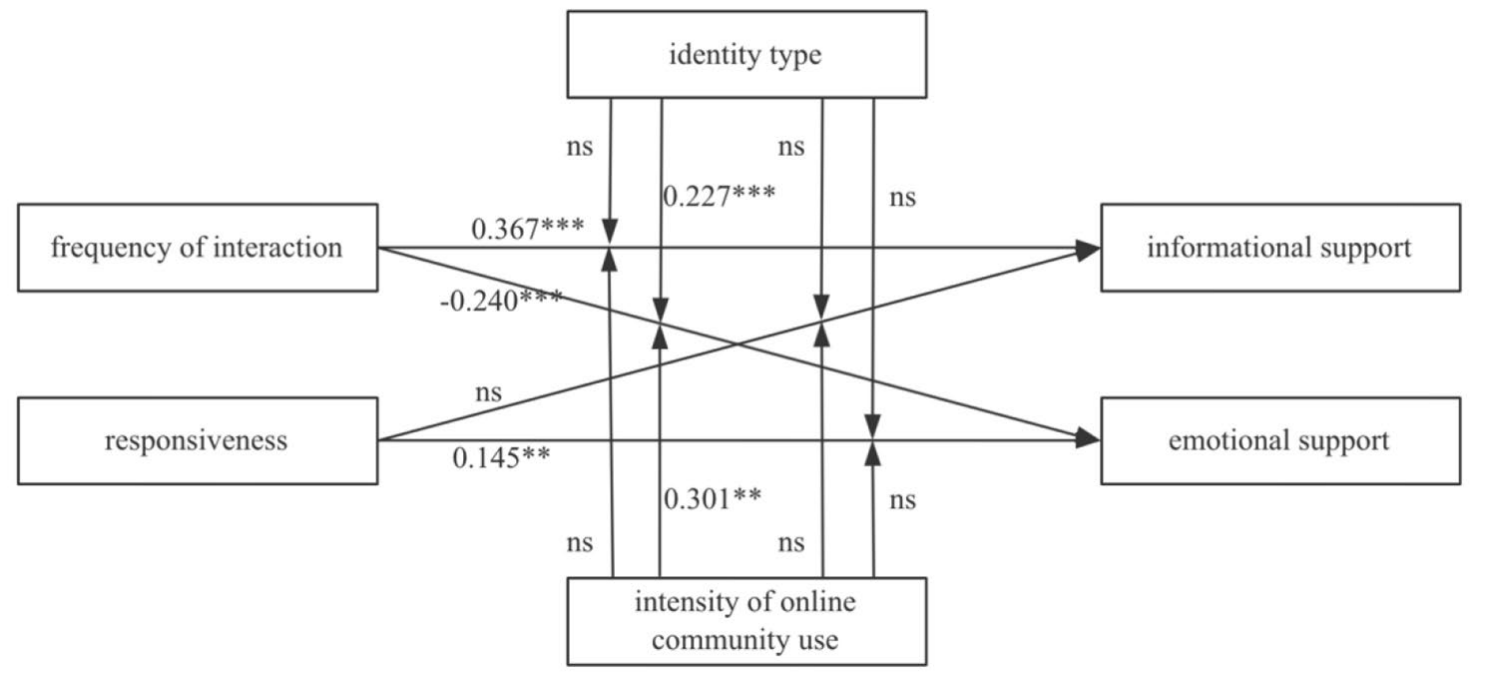
The findings for H1 to H4

Hierarchical regression analysis was performed. Informational support and emotional support were treated as dependent variables, the frequency of interaction and responsiveness were treated as independent variables, and the help seeker’s identity type and intensity of online community use were treated as moderating variables. The interactions between the independent variables and moderating variables were incorporated into the regression analysis to examine the effect of each moderating variable. The results of the regression analysis are shown in Table 2, and the findings for H1 to H4 are shown in Fig. 2.

**Table 2:** Results of the regression analysis

	Informational support				Emotional support			
	Model 1		Model 2		Model 3		Model 4	
	β	t	β	t	β	t	β	t
Intercept	-0.001	-0.028	-0.008	-0.157	0.005	0.094	0.012	0.228
Frequency of interaction	0.367	7.222***	0.345	6.611***	-0.240	-4.642***	-0.246	-4.642***
Responsiveness	-0.039	-0.741	-0.024	-0.472	0.145	2.726**	0.124	2.388*
Help seeker’s identity type	0.218	4.331***			0.029	0.559		
Frequency of interaction × help seeker’s identity type	-0.017	-0.344			0.227	4.441***		
Responsiveness × help seeker’s identity type	-0.046	-0.616			-0.029	-0.387		
Intensity of online community use			-0.141	-1.502			0.247	2.596*
Frequency of interaction ×Intensity of online community use			-0.150	-1.886			0.301	2.678**
Responsiveness × Intensity of online community use			0.094	0.693			-0.146	-1.056
R2	0.585		0.646		0.558		0.520	
ΔR2	0.592		0.675		0.571		0.607	
F	15.026***		11.338***		8.873***		9.071***	

Notes: *p < 0.05,**p < 0.01, ***p < 0.001

### Results analysis of informational support

For Model 1, the regression coefficient of the frequency of interaction was significant (β = 0.367, p < 0.001), indicating that the frequency of interaction positively impacts informational support; the regression coefficient of responsiveness was not statistically significant. As such, hypothesis H1a is supported while H2a is not.

The results of hypothesis H1a indicate that frequent information interaction helps users receive informational support. In the Weibo COVID subforum, help seekers posted messages in accordance with the standardized formats specified by the administrator of Sina Weibo, and the messages only concisely outlined the issues for which help was asked. Detailed background information and specific descriptions of the help seekers’ issues were not included in the original help-seeking post. As such, help seekers interacted with other users in the comments section under the original help-seeking post to provide more detailed information about the help requested, such as what approaches had already been attempted and the difficulties the help seeker was still facing. In the interaction between the help seeker and other users, the help seeker usually provides detailed help-seeking information so that other users can more accurately provide the information and assistance the help seeker needs. It is commonly held that the interaction among users within online community fosters trust among community members to [33]. Therefore, the weak connection between the help seeker and other users is strengthened, and the degree of trust between the help seeker and other users is also increased, which prompts other users to provide more support and assistance to the help seeker.

Hypothesis H2a is not supported, which reveals that timely responses by other users did not enhance the help seeker’s informational support acquisition during the interactive process. The timely reply by other users indicates that they paid attention to or responded to the help-seeker’s help-seeking information in a timely manner. Even so, the responders may not be able to provide the information, resources, or methods to address the help-seeker’s difficulties; that is, they cannot provide information support for help-seekers.

In addition, in Model 1 and Model 2, the influence of the help seeker’s identity type and intensity of online community use on informational support were investigated, and it was found that the help seeker’s identity type has a positive impact on the help seeker’s receipt of informational support in online communities. However, the help seeker’s intensity of online community use had no significant influence on the help seeker’s receipt of informational support. Furthermore, the effect of the help seeker’s identity type and the help seeker’s intensity of online community use as the moderating variables of the relationship between the frequency of interaction and responsiveness and informational support were examined in Model 1 and Model 2, respectively. Neither of the results was statistically significant. Therefore, hypotheses H3a, H3c, H4a, and H4c are not supported.

The results of Model 1 and Model 2 show that the relationship between the frequency of interaction and responsiveness and informational support was not affected by the help seeker’s identity type and the intensity of online community use. In the COVID-19 pandemic, whether help seekers can obtain more informational support by posting help-seeking messages depends mainly on the frequency of interaction between the help seeker and others and the help seeker’s identity type. If the help seeker can maintain a high frequency of interaction with other users or the help seeker’s influence on Weibo is relatively high, he/she can obtain more informational support.

### Results analysis of emotional support

For Model 3, the regression coefficients of the frequency of interaction and responsiveness were both statistically significant, but frequency of interaction negatively impacts the emotional support received by the help seeker (β=-0.240, p < 0.001); additionally, responsiveness positively impacts the emotional support (β = 0.145, p < 0.01). As such, H1b is not supported, and H2b is supported.

It may be puzzling that hypothesis H1b is not supported. After all, it is not consistent with the conclusions of previous related studies. However, after scrutiny, we believe that it is possible that the more frequent the interaction is, the less emotional support received by help seekers, especially in public health emergencies. As proposed by Chen et al. [34] and Lin and Kishore [13], under usual circumstances, information interaction facilitates online community users to receive emotional support to a certain extent. However, during the COVID-19 pandemic, in the high-frequency interactions between help seekers and other users, the predicaments help seekers face could be displayed, and negative emotions such as sadness and despair could be revealed. The mood of users in online communities was negativeand reflected by panic, pessimism, and blindly following others’ actions. Furthermore, incited by various types of information, the mentalities of users may resonate. Amidst the high frequency of information interaction during the COVID-19 pandemic, negative emotional information intertwined, and the receipt of emotional support by help seekers was low.

Hypothesis H2b is supported, which indicates that the timely response of other users is conducive to help seekers’ emotional support acquisition. We note that although prompt responses to the help seeker do not always result in more informational support, they still constitute psychological comfort and emotional support to help seekers.

In Model 3 and Model 4, the influence of the help seeker’s identity type and the help seeker’s intensity of online community use on emotional support were explored. The results show that the help seeker’s intensity of online community use positively impacts the help seeker’s emotional support acquisition and that the help seeker’s identity type has no significant influence on the emotional support acquisition. Help seekers who have a high intensity of online community use are more likely to obtain emotional support.

Furthermore, the effect of the help seeker’s identity type and intensity of online community use as moderating variables of the relationship between the frequency of interaction and responsiveness and emotional support were examined. For Model 3, the interaction term between the frequency of interaction and the help seeker’s identity type had a positive predictive effect on emotional support (β = 0.227, p < 0.001); for Model 4, the interaction term between the frequency of interaction and the help seeker’s intensity of online community use had a positive predictive effect on emotional support (β = 0.301, p < 0.01). However, the result regarding the help seeker’s identity type and intensity of online community use as moderating variables of the relationship between responsiveness and emotional support is not significant. Therefore, H3b and H4b are supported, and H3d and H4d are not supported.

### Testing of moderating effects

**Fig. 3:**
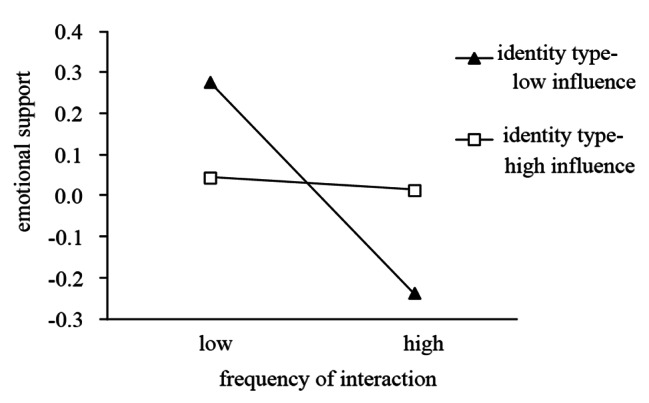
Interaction effect for frequency of interaction and identity type on emotional support

**Fig. 4:**
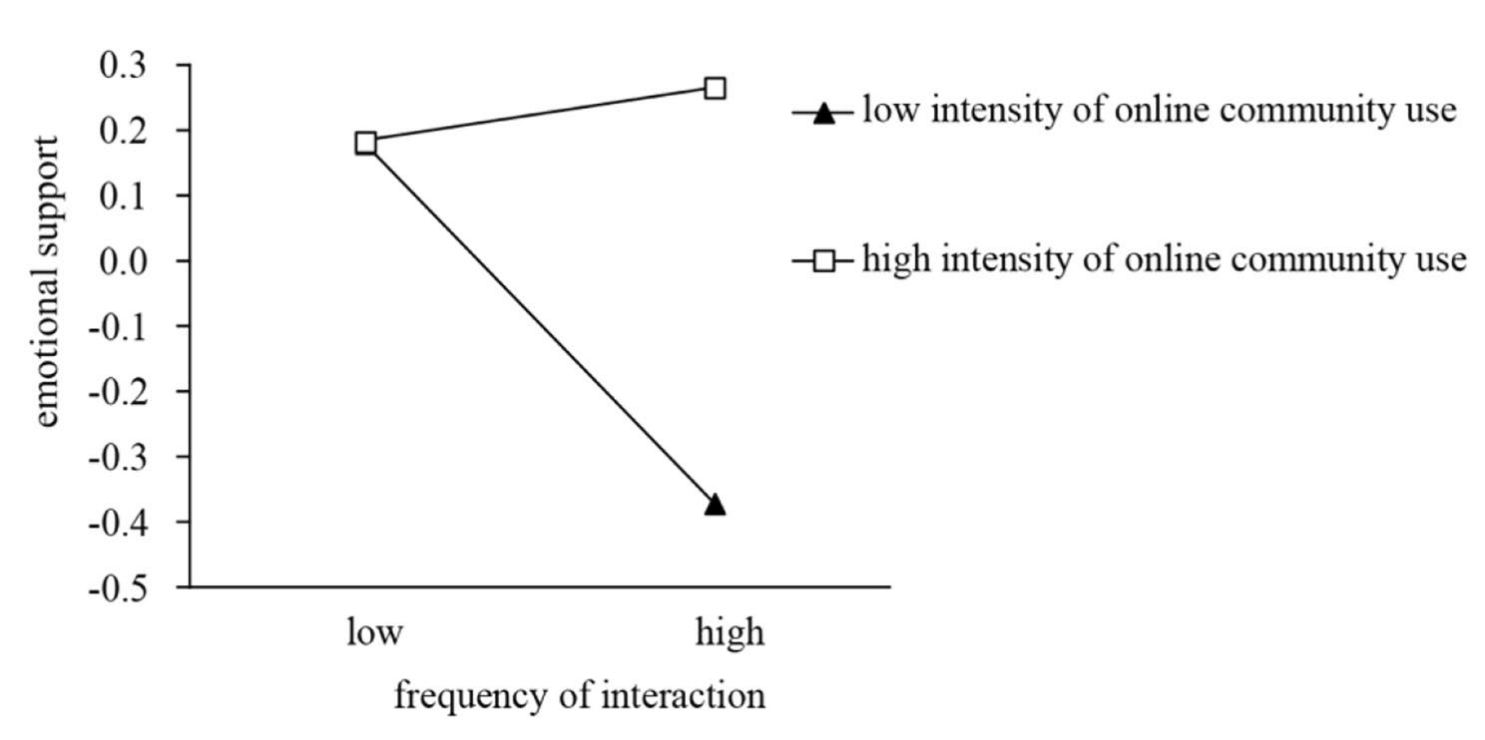
Interaction effect for frequency of interaction and intensity of online community use on emotional support

To reveal how the help seeker’s identity type moderates the relationship between the frequency of interaction and emotional support, a simple effects test was performed. As in other studies [35, 36], the Johnson-Neyman method was performed for the simple slope test. Simple slope graphs were created, as shown in Figs. 3 and 4. Figure 3 indicates that when a help seeker has a low influence on Weibo, the frequency of interaction negatively impacts emotional support (β=-0.464, p < 0.001), and this negative impact weakens as the help seeker’s influence on Weibo increases. Figure 4 indicates that when a help seeker had a higher intensity of online community use, with the increase in the frequency of interaction, emotional support did not change significantly (β = 0.050, p > 0.05). When the help seeker had a lower intensity of online community use, the frequency of interaction negatively impacts emotional support; the higher the frequency of interaction is, the stronger the negative impact becomes (β=-0.440, p < 0.001).

Notably, when help seekers post messages for help during the COVID-19 pandemic, the receipt of emotional support varies significantly with the characteristics of the help seeker. In the Weibo COVID subforum, many users with high influence on Weibo posted help-seeking information on behalf of other help-seekers; in these cases, there was more neutral content in the interactions, and the users with high influence on Weibo did not experience significant changes in emotional support acquisition. In addition, compared with help seekers with low intensity of online community use who obtained less emotional support in more interactions, the emotional support acquisition of help seekers with high intensity of online community use is less affected, even if there is a large amount of negative information regarding unoptimistic conditions and dilemmas in the information interaction.

### Robustness tests

To examine the robustness of the research results, help seeker’s identity type and intensity of online community use were not used as moderator variables, but the data were divided into two subsamples respectively according to the categories of help seeker’s identity type (low influence and high influence) and intensity of online community use (low intensity and high intensity); regression analysis was then performed by the least-squares method. The purpose of this analysis was to verify whether the frequency of interaction and responsiveness have a significant impact on informational support and emotional support without the help seeker’s identity type and intensity of online community being used as moderator variables. The results of the robustness tests are shown in Tables 3 and 4. The results are consistent with the results of the regression analysis. This finding demonstrates that the hypothetical results described above are still obtained without the moderator variables.

**Table 3:** Parameter estimates in the robustness analysis of informational support

Variable	Informational support		Informational support	
	High influence	Low influence	High intensity	Low intensity
Constant	0.553 (4.63)***	0.114 (0.49)	0.0148 (0.24)	1.113 (1.16)
Frequency of interaction	0.396 (3.41)***	0.349 (7.43)***	0.562 (3.18)***	0.347 (6.51)***
Responsiveness	-0.0129 (-0.12)	-0.021 (-0.44)	-0.065 (-0.59)	− 0.034 (-0.59)
ΔR2	0.200	0.205	0.116	0.13
F	11.94	18.57	3.4	15.01

**Table 4:** Parameter estimates in the robustness analysis of emotional support

Variable	Emotional support		Emotional support	
	High influence	Low influence	High intensity	Low intensity
Constant	0.116 (1.17)	0.142 (0.48)	-0.02 (-0.54)	1.626 (1.58)
Frequency of interaction	-0.147 (1.52)*	-0.433 (-7.29)***	-0.162 (-1.51)*	-0.301 (-5.26)***
Responsiveness	0.135 (1.52)*	0.145 (2.38)**	0.236 (3.46)***	0.108 (1.72)*
ΔR2	0.021	0.222	0.148	0.100
F	1.95	20.52	4.19	11.37

## Discussion

### Summary of findings

The influence of the frequency of interaction and responsiveness on obtaining online social support for help seekers was analyzed, and the moderating effect of the help seeker’s identity type and online community use intensity on the relationship between information interaction and online social support was explored.

The results show that the frequency of interaction between the help seeker and others positively impacts the help seeker’s acquisition of informational support but negatively impacts the acquisition of emotional support; the responsiveness (of other users) has a positive effect on the acquisition of emotional support. The help seeker’s identity type positively impacts the acquisition of informational support, and the help seeker’s intensity of online community use positively impacts the acquisition of emotional support. Moreover, the help seeker’s identity type and intensity of online community use have a significant moderating effect on the relationship between the frequency of interaction and the emotional support obtained by the help seeker.

During the COVID-19 pandemic, frequent information interaction between help seekers and other users benefits the help seeker’s receipt of informational support and improves the efficiency of the help seeker’s information acquisition, and timely responses from other users are more conducive to help seekers receiving emotional support. Furthermore, when help seekers post messages for help, the receipt of emotional support varies significantly with the characteristics of the help seeker. Although the higher the frequency of interaction is, the lower the acquisition of emotional support is, the less affected the acquisition of emotional support of help seekers with high influence in the online community or high intensity of online community use. They are more familiar with the information interaction patterns and characteristics in online communities and can regard the content of information interaction in online communities more rationally.

### Implications

This study contributes to the existing literature by systematically analyzing help-seeking on Sina Weibo during the COVID-19 pandemic, which further enriches and extends the theoretical framework and empirical research on help-seeking in online communities during public health emergencies. Hypotheses regarding the influence of the frequency of interaction and responsiveness on obtaining online social support were tested, and the moderating effect of help seekers’ identity type and intensity of online community use was also tested.

Moreover, information interaction in online communities during public health emergencies is an indispensable channel for users to obtain help. This study analyzes the impact of user information interaction on obtaining help-seeking information from online communities for social support. Although previous research has paid much attention to help-seeking behavior in online communities, this study goes beyond the extant studies focusing on understanding the complex information interaction mechanisms in the context of public health emergencies.

Furthermore, the research model, its constructs, and the measurement of the constructs proposed in this research are expected to be basic measuring methods that can be applied to information interaction within the help-seeking context, which provides a mixed-method approach and cross-cultural perspectives for future related research.

The findings of this study will serve as a reference for users to obtain the help they need during public health emergencies, for online communities to provide better information dissemination services, and for government and charity organizations to more effectively provide assistance.

For individual help seekers, frequent information interaction with other users facilitates receiving informational support; furthermore, for individual help seekers, having users with a higher influence in the online community post or repost messages on their behalf while frequently interacting with other users in the comments section under the original help-seeking post, will assist the help-seeking information to spread more widely. Furthermore, for individual help seekers, a high intensity of online community use is conducive to receiving emotional support.

Online community operators must pay more attention to the design of information interaction functions to not only make it easy for users to find help-seeking channels but also facilitate the acquisition of informational support by help seekers. For example, during the COVID-19 pandemic, information about hospitals and hospital beds, disease and drug information, and measures to control the spread of the virus were collected.

The implication for government organizations and charity organizations potentially involves implementing the following measures to capitalize on the information functions of online communities: collect help-seeking information promptly; sort and forward it to government departments/charity organizations accordingly; and actively and promptly respond to help seekers. In short, government and charity organizations should provide help seekers with social support through refined management.

### Limitations

This study explores the impact of individual help seekers’ information interactions in online communities on the acquisition of online social support during a public health emergency. Despite all of the work that was done in this study, there are certain limitations. For one thing, for help-seeking information interactions, a more detailed analysis should be conducted based on the content feature of help-seeking information or the help-seeking condition. Another issue is that Weibo is just one representative of the many platforms of online communities. There are other online community platforms for help-seeking, such as Zhihu or WeChat. Data from different communities would not lead to significantly different results. However, the same factors may affect the degree of social support acquisition differently in various communities, which is also worth exploring. The aforementioned issues deserve further exploration in the future.

## Conclusions

By investigating help-seeking posts on Sina Weibo during the COVID-19 pandemic, we believe that the frequency of interuser interaction positively impacts informational support and negatively impacts emotional support and that responsiveness positively impacts emotional support. We also found that the help seeker’s identity type and intensity of online community use have significant moderating effects. We hold the opinion that enhancing the frequency of information interaction helps with the receipt of informational support, and timely responses from other users are more helpful for help seekers to receive emotional support. In this study, online community users and operators as well as governments and charity organizations can reference our study to guide their behaviors, operations, or decisions.

## Data Availability

The datasets used and analyzed during the current study are available from the corresponding author on reasonable request.
